# The Paradox of Interactive Media: The Potential for Video Games and Virtual Reality as Tools for Violence Prevention

**DOI:** 10.3389/fcomm.2020.580965

**Published:** 2020-11-23

**Authors:** Nicholas David Bowman, Sun Joo Ahn, Laura M. Mercer Kollar

**Affiliations:** 1 College of Media and Communication, Texas Tech University, Lubbock, TX, United States,; 2 Grady College of Journalism and Mass Communication, University of Georgia, Athens, GA, United States,; 3 Division of Violence Prevention, National Center for Injury Prevention and Control, Centers for Disease Control and Prevention, Atlanta, GA, United States

**Keywords:** video games, virtual reality, violence prevention, intervention, media violence

## Abstract

Interactive media such as video games and virtual reality (VR) provide users with lived experiences that may be dangerous or even impossible in daily life. By providing interactive experiences in highly authentic, detail-rich contexts, these technologies have demonstrated measurable success in impacting how people think, feel, and behave in the physical world. At the same time, violent interactive media content has been historically connected with a range of antisocial effects in both popular press and academic research. Extant literature has established a small-but-statistically significant effect of interactive media violence on aggressive thoughts and behaviors, which could serve as a risk factor for interpersonal violence. However, left unexplored is the seemingly paradoxical claim that under some conditions, interactive media experiences might protect against interpersonal violence. Drawing on advances in media theory and research and the evolution of interactive media content and production practices, the current manuscript suggests ways in which interactive media violence may be leveraged to lower the likelihood of real-world violence experiences. For example, research on both violent and non-violent games has found that players can (a) express guilt after committing violent acts, (b) report reflective and introspective emotional reactions during gameplay, and (c) debate the morality of their actions with others. Regarding VR, studies have demonstrated that (a) witnessing physical violence in immersive spaces led participants to take the perspective of victims and better understand their emotional state and (b) controlled exposure to traumatic or violent events can be used for treatment. Broadly, studies into video games and VR demonstrate that the impact of actions in virtual worlds transfer into the physical worlds to influence (later) attitudes and behaviors. Thus, how these experiences may be potentially harnessed for social change is a compelling and open consideration, as are side-effects of such interventions on vulnerable groups. The current manuscript summarizes emerging research perspectives (as well as their limitations) to offer insight into the potential for interactive media violence to protect against real-world violence victimization and perpetration.

## INTRODUCTION

Violence, such as interpersonal violence, is preventable, has lasting impacts on physical and mental health, and is among the leading global causes of death and injury (World Health Organization, 2002, 2014). Although not directly connected to public health models, media psychologists often study violence as portrayed in mass media as a potential cause or correlate of violence. Meta-analyses report statistically significant but overall small effects for both passively viewed violence (such as that featured in films and on television; Anderson et al., 2017) and interactive violence (where media users perpetrate violent acts, such as in video games; Calvert et al., 2017) in mediated content on some forms of aggression, such as aggressive thoughts and feelings and some retributive behavior.

In our essay, we acknowledge the extant empirical record associating media violence with some forms of aggression and seek to explore future and emerging research paths based on recent advances in media psychology (Oliver and Raney, 2011; Oliver et al., 2015; Hemenover and Bowman, 2018; Tamborini et al., 2018). These advancements support the seemingly paradoxical claim that exposure to mediated violence, especially through environments in which one has to both perpetrate violence and witness their actions and aftermath in rich contextual details, may potentially influence perceptions and behaviors that serve as protective factors for reducing interpersonal violence by influencing how players perceive, understand, and respond to violence.

We explore this potential by first defining violence broadly and within the context of media (including the notion of interactive media violence), and then exploring past work associating mediated violence with aggression and related constructs. From this, we present emerging theory and data from two interactive media forms that suggest interactive media violence could be a key leverage point for violence prevention: video games and virtual reality (VR). The paper concludes with suggestions for future research by expanding the scope of violence prevention programs to consider the use of interactive media violence that can be safely simulated in gaming and VR applications.

## VIOLENCE AND VIOLENCE PREVENTION

Violence is defined as the intentional use of physical force or power, threatened or actual, against oneself, another person, or against a group or community that either results in or has a high likelihood of resulting in injury, death, psychological harm, maldevelopment, or deprivation (Krug et al., 2002). Interpersonal violence (including child abuse and neglect, youth violence, intimate partner violence, sexual violence, and elder abuse) is a leading cause of death and injury in the United States (Sumner et al., 2015) and globally (World Health Organization, 2002, 2014). Violence has lasting impacts on health, spanning injury, disease outcomes, risk behaviors, maternal and child health, and mental health problems.

**FIGURE 1 |:**
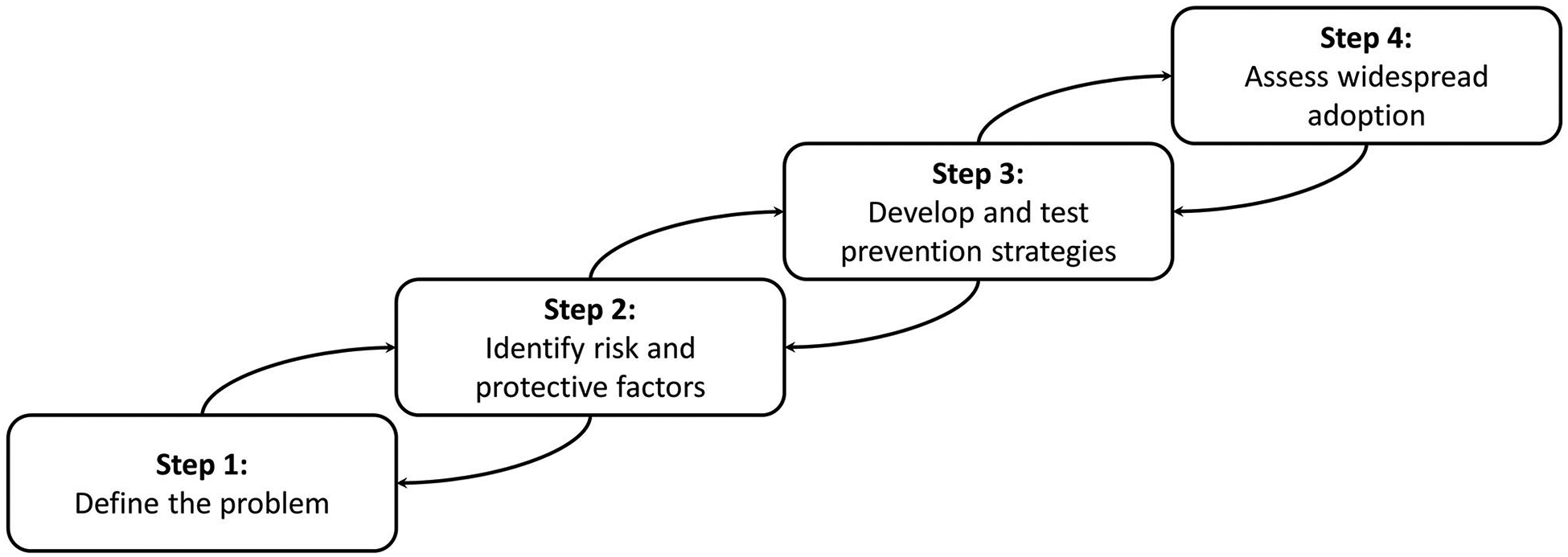
A public health approach to violence prevention, adapted from the US Centers for Disease Control and Prevention. Note: Violence prevention strategies start with defining and understanding the problem. At each step of the model, previous work is consulted as part of formative assessment practices.

**FIGURE 2 |:**
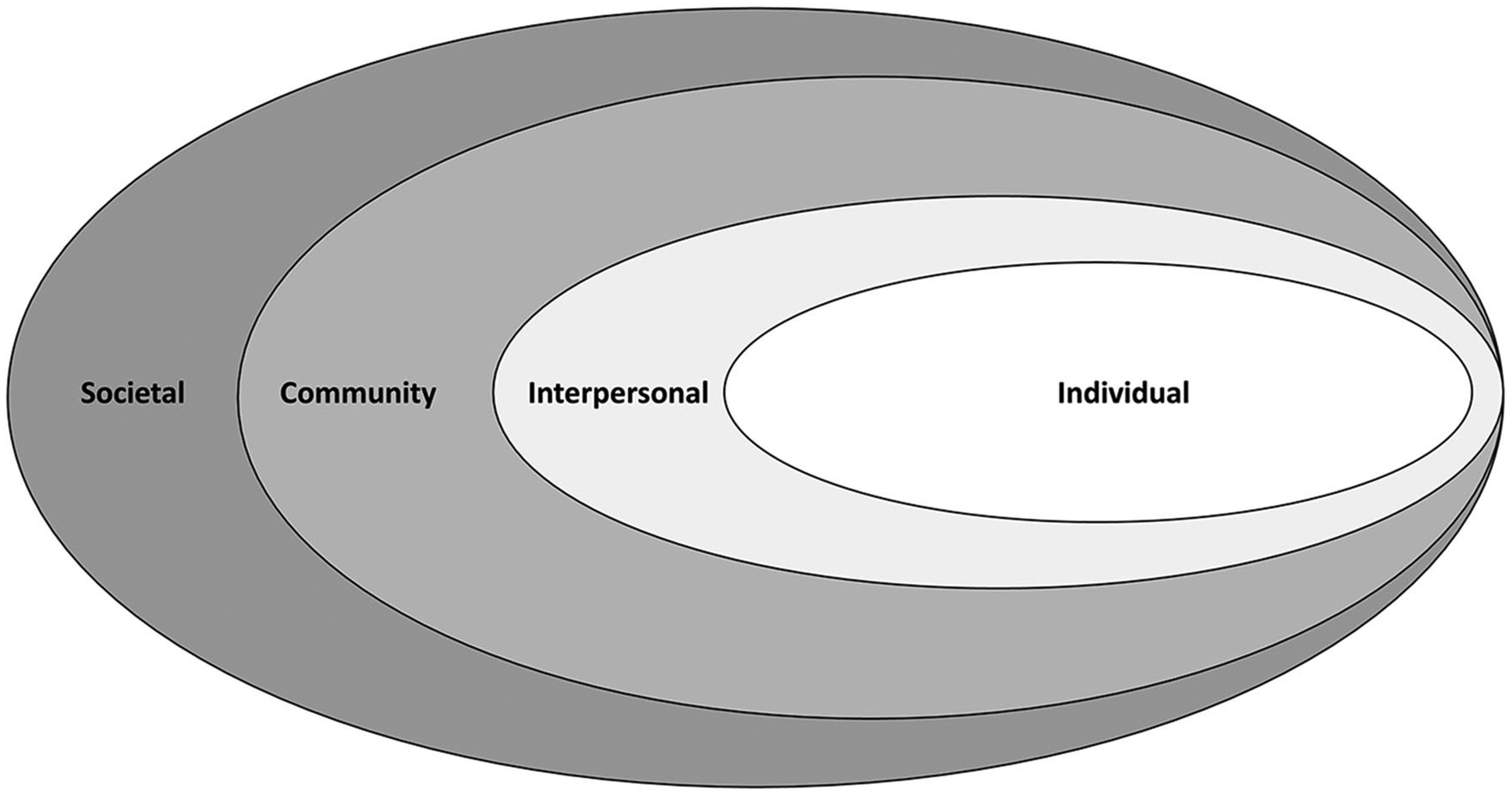
Social-ecological model of violence prevention (from Centers for Disease Control and Prevention, 2020b).

Violence is preventable using a public health approach. This approach follows a common four-step process (see Figure 1; Centers for Disease Control and Prevention, 2020a). Briefly summarized from the Centers for Disease Control and Prevention (2020a), the first step of the public health approach is to define and monitor a given type of violence, which usually involves defining and explicating violence and then assessing descriptive data about said violence. The second step includes a focus on identifying risk and protective factors, understood respectively as characteristics that increase or decrease the odds of violence—critically here, public health approaches are less focused on identifying specific “causes” of violence but rather, understanding the characteristics of a given scenario that influence the likelihood that one experiences violence. There is also recognition that risk and protective factors for one form of violence impact other forms of violence (Wilkins et al., 2014). At the third step, prevention strategies are developed and rigorously tested to determine their efficacy for violence prevention—such strategies might be aimed at either reducing risk factors or encouraging protective factors, and often involve a combination of both. Finally, at the fourth step, strategies shown to be effective in step three are disseminated and implemented broadly. While steps may occur sequentially, the process is cyclical, and steps may be revisited at any point. Overall, this public health approach offers a framework for asking and answering questions to build successful violence prevention efforts. Although violence prevention includes primary, secondary, and tertiary prevention, CDC’s violence prevention efforts are focused on *primary prevention*, or stopping violence before it starts. These prevention efforts are often, although not exclusively, guided by the social-ecological model (Centers for Disease Control and Prevention, 2020b; Figure 2), which presumes that any health interventions (such as violence prevention initiatives) must be understood within individual (e.g., attitudes, beliefs, and behaviors), interpersonal (e.g., social support), community (e.g., school or work environment), and broader social contexts (e.g., social and cultural norms, public policy; see “risk and protective factors” on CDC/Division of Violence Prevention’s website: www.cdc.gov/violenceprevention). The model also helps organize and identify a range of factors that may increase or decrease risk of experiencing violence.

Successful and empirically validated strategies of the best available evidence for violence prevention are laid out in the CDC’s Division of Violence Prevention’s five technical packages (Centers for Disease Control and Prevention, 2018), focused on child abuse and neglect, intimate partner violence, sexual violence, suicide, and youth violence. Each technical package includes three main components: strategies (overview of actions required to prevent a given form of violence), approaches (specific programs, policies, or practices to advance the strategy), and evidence (empirical support for the suggested approaches). Additionally, there is an *Adverse Childhood Experiences* resource document that compiles information from all technical packages and literature (Centers for Disease Control and Prevention, 2019). While available evidence provides the use of media and technology as delivery methods and media campaigns as a community-level approach to violence prevention, little is known how interactive media may serve as a violence prevention approach or influence protective factors.

## MEDIA VIOLENCE AND INTERACTIVE MEDIA

We can understand interactive media violence through the broad lens of media effects research. There is no universal definition of media effects, but we can generally understand the research perspective as having a discrete focus on how mediated communication—anything from printed books and television shows to video games and VR—impacts the end user’s thoughts, feelings, and actions. Rutledge (2013) offers a similar definition of the emerging field of media psychology, focused on the “complex relationship between humans and the evolving [technological] environment” (p. 43). Similar logic was proposed decades earlier by Klapper (1960), who suggested that media effects occur “among and through a nexus of mediating factors and influences” (Klapper, 1960, p. 8). Oliver et al. (2018) assert that media effects research tends to focus on the deleterious effects of media, and Lowery and DeFleur (1994) overview a history of media effects research based on a “hypodermic needle” model by which content was presumed to have a direct, powerful, and universal influence on audiences (Neuman and Guggenheim, 2011). Contemporary approaches to media effects research encourage a more functional approach (Bowman, 2019a) that unpacks (1) media’s broad uses, (2) media effects, and (3) dynamic interactions between users and media (also discussed in Neuman and Guggenheim, 2011).

Given the volume of studies focused on media violence, meta-analysis techniques are commonly used to quantify effects. For example, Anderson and Bushman (2002) found statistically significant summary main effects of non-interactive violent media content on aggressive behavior, ranging from about *r* ~ 0.15 to *r* ~ 0.25 depending on the type of study being conducted (i.e., longitudinal studies showing weakest and laboratory studies showing strongest effects). When correcting for alleged publication biases in extant literature, Ferguson and Kilburn (2009) report a smaller-yet-still-significant summary effect of media violence on aggression depending on the medium (e.g., their sub-analysis of non-interactive media reported effect sizes of *r* = 0.04 for television content and *r* = 0.10 for films). Their study also found variance in effects depending on whether or not effects were found using *ad hoc* reports of aggression (*r* = 0.25) compared to observed aggressive behavior (*r* = 0.08). Other studies have assessed violence using a variety of laboratory-based methods for assessing aggressive thoughts, feelings, and implied or explicit harmful behaviors (McCarthy and Elson, 2018). Savage and Yancey (2008) challenged whether or not media violence impacts criminal violence—actions of violence that would violate criminal codes—as their analysis yielded a non-significant summary effect, *r* = 0.057 (95% CI −0.006 to 0.119); Ferguson and Kilburn (2009) found a similar non-significant effect (*r* = 0.02, −0.12 to 0.16) between media violence exposure and violent criminal behavior.

Similar meta-analytic results have been observed for interactive media violence, usually focused on video games. The qualifier *interactive* here refers to the user’s ability to alter the form or content of the mediated experience (Steuer, 1992), which was thought to be particularly relevant to violent content. Instead of the user passively and innocently witnessing on-screen violence, interactive media has the user play a direct role in perpetrating those acts. In the face of intense debates regarding video game violence (de Vrieze, 2018), the American Psychological Association convened a task force to summarize this literature (Calvert et al., 2017). That group found that although violent video games were not a risk factor in criminal or delinquent behavior (as reported above, with non-interactive media), small-but-significant associations were found between gaming and feelings of aggression, increased arousal, and violent ideation [although recent work from Ferguson et al. (2020), was unable to reproduce the effect size magnitudes from that task force report]. Recent meta-analytic work by Prescott et al. (2018) that focused only on longitudinal studies involving violent games and acts of “overt, physical aggression” (p. 9,982) again reported small-but-significant overall effects ranging from β = 0.078 to β = 0.113. Most importantly for the current manuscript is the work of Mathur and VanderWeele (2019), who found evidence of convergence between different meta-analyses (including the above-mentioned Prescott analysis and studies examined by Calvert et al. (2017) supporting the twin claims that (a) interactive media violence can cause aggression in users, and (b) these effects are overall quite small (with nearly no studies reporting effect sizes larger than 4% of explained variance in aggression). Finally, a paucity of work on VR-based interactive violence on aggression find results similar to those reported here—somewhat unsurprising, as these earlier studies tended to focus on VR video games (see Persky and Blascovich, 2006, 2007).

While not challenging the extant literature on violent content and aggressive outcomes, we respectfully suggest that much of this work has myopically focused on presumed negative effects of content without a deeper elaboration of how that content is actively consumed and understood by users. As was suggested by Klapper (1960), media audiences actively engage with and make-sense of on-screen content and thus, a narrow focus on the content alone is insufficient to understand media effects. Applied to interactive media, Wellenreiter (2015) argues that on-screen content is inherently dynamic and co-authored as both the user and the system combined to create, interpret, and engage the system in ways that are somewhat unique to each user. Schmierbach (2009) suggests that this co-authorship poses a challenge for researchers attempting to quantify (for example) video game violence, as the amounts of and meaning behind violent acts will change depending on how the player engages the game. As will be argued for the balance of this manuscript, some of the same violent content shown to encourage aggression in users has also been shown to encourage unprompted moral debate (Malazita and Jenkins, 2017) and moral reappraisal (Tamborini et al., 2018), feelings of guilt (Grizzard et al., 2014), perspective-taking (Seinfeld et al., 2018; de Borst et al., 2020), as well as a broader feeling of eudaimonia and meaningfulness from a variety of video games (including games with overt as well as mild forms of violence; Oliver et al., 2015; Rogers et al., 2016). From this perspective, the following sections propose emerging theory and logic for how at least two interactive technologies—video games and VR—have the potential to positively influence how users perceive, understand, and respond to violence, both in the digital and the physical world.

## VIDEO GAMES AND VIOLENCE

Perhaps the “original exemplar” of interactive media violence, video games have been historically connected to aggressive and combative themes. One of the earliest video games, *Spacewar!*, invoked military themes reminiscent of the Cold War between the United States and the Union of Soviet Socialist Republics in the middle 20th century (Graetz, 1981)—a game locking two players piloting different ships (the “needle” or the “wedge”) in mortal combat while being pulled into the gravitational well of a central star. Later games such as *Pong* and *Space Invaders* likewise featured competition—the former pitting players against each other, the latter again simulating war-like themes. Indeed, even contemporary research into video games suggests challenge and competition to be among the most prevalent motivations for playing games in the first place (Sherry et al., 2006; Yee, 2006).

Importantly, these early games were mostly non-violent in nature—at least, they did not feature elements of unjustified, graphic, or realistic violence that signal violence perpetration (Tamborini et al., 2013). Kocurek (2012) argues that the 1976 release of *Death Race* marked a watershed moment in the public perception of video games, as it was the first game that received widespread attention specifically for being violent. The game, which was loosely inspired by the 1975 science fiction film *Death Race 2000* (which featured elaborated and gory vehicular manslaughter as a central plot device), tasked players with piloting their own race car around a blank field, chasing “gremlins” around the screen that were roughly anthropomorphic stick figures. Just as in the movie, players are awarded points for running down and eliminating the on-screen “gremlins” —each elimination converts the “gremlin” to a tombstone, which impedes future driving paths. The game’s controls were also designed to mimic a physical car, including a realistic steering wheel, gear shifter, and gas and brake pedals (referred to as natural mapping by technology scholars, see Skalski et al., 2011). In an interview with The *New York Times*, a behavioral psychologist with the National Safety Council stated that:

“Nearly 9,000 pedestrians were killed last year [by vehicles], and that’s no joke… On TV, violence is passive. In this game a player takes the first step in to creating violence … I shudder to think what will come next if this is encouraged. It’ll be pretty gory.”

(Driessen, as cited by Blumenthal, 1976).

The debate around *Death Race* “forged a strong tie between video gaming and violence in the public imagination” (Kocurek, 2012; para. 1), which catalyzed moral panics associated with interactive media violence (Bowman, 2016). Similar markers in the violent video game timeline include the so-called “Mortal Monday” (September 13, 1993) release of *Mortal Kombat* for home gaming consoles—a game featuring hand-to-hand combat in which victorious players were given the chance to execute their opponents with over-the-top “fatalities” (such as one player ripping the still-beating heart out of the others). *Mortal Kombat* was a lightning rod for controversy, resulting in numerous US Congressional hearings and, eventually, the creation of the Entertainment Software Rating Board system for rating the content of video games (Andrews, 1993). The 1990s also saw controversy over “first-person shooter” video games in which the player’s primary objective was to use weapons to search and kill other characters, with critics labeling these games “murder simulators” (Silverman, 2007). In the 2000s, the *Grand Theft Auto* series were heavily scrutinized for permitting and even encouraging physical, weapon-based, and vehicular violence—even more so, folding this violence in with misogynistic, racist, and other socially deleterious themes (Bowman, 2014a,b). Games in the *Grand Theft Auto* series commonly faced restriction on their sales (often by being rated *M* for mature audiences), although the series’ latest game *Grand Theft Auto V* sold a record 110 million copies as of May 2019 (Kain, 2019).

### Bringing Context in the Discussion of Violence in Contemporary Video Games

Of course, not all video games are violent ones and perhaps most critically for the current discussion, not all violent video games celebrate violence. Benedetti (2010) wrote that were “growing up” along with their audience—by 2018, the average age of a video gamer in the United States was 34, and over 70% were over the age of 18 (Entertainment Software Association, 2018). Along with this, she observed that video games were beginning to “peer into the dark reaches of the very real human heart to deliver stories that are thrilling, chilling and utterly absorbing” (para. 6). Designers such as Schell (2013) explained that “[just as] film wasn’t taken seriously as a medium until it learned to talk, games are waiting to learn to listen.” Going further, he talked about video games as having evolved to focus on “*above-the-neck”* verbs (such as talking, asking, and pleading; notions associated with emotional and social concerns) alongside their already strong focus on “below-the-neck” verbs (action orientations such as running, jumping, and fighting).

Both Stober (2004) and Bowman (2019b) suggest that this evolution of video games toward having more serious, pensive, and reflective content follows a more generalized pattern seen in past forms of media entertainment—as communication technologies evolve, their content moves from more basic technological demonstration toward more innovative and unique ways of storytelling. Williams (2013) explained the rationale behind his design of *Spec Ops: The Line*. As a military themed game, there is a heavy emphasis on warfare and weapons-based combat, common to many third- and first-person shooter games. What made *Spec Ops: The Line* unique was the way the game contextualized the on-screen violence. For example, in a pivotal scene in which the game’s main character Walker is facing heavy fire, he elects to release a white phosphorus canister (a chemical weapon) on opposing forces, and despite the pleas of his fellow soldiers. As the player navigates the remnants of the battlefield, they are forced to confront the atrocities of chemical war. Indeed, the closing scene of two corpses—a mother clutching her daughter while both are burned nearly beyond recognition—was criticized by gaming journalists for its gruesome portrayals as well as the fact that the game “forced” players to commit war crimes as part of gameplay (Roberts, 2014). In response, Williams (2013) explained that the game was designed to *contextualize* rather than glorify war, as the narrative that unfolds from this scene follows Walker’s slow mental decline in the face of having to reconcile a series of seemingly impossible moral quandaries involving gruesome acts of war. Another example can be found in *Call of Duty: Modern Warfare 2* in which players found themselves inserted into a terrorist cell bent on massacring civilians in an airport. The terrorists unleash waves of gunfire on an innocent population, and the player has only two options: shoot civilians or watch helplessly as the other terrorists do the same. Facing critique for this level design, the game’s writers explained that their purpose was to “make the player feel anything at all” (Totilo, 2012). Notably, when directly comparing the two games, *Call of Duty*’s scenario was critiqued for being superfluous and unnecessarily gratuitous—even during playtesting, many objected to the levels’ content and some refused to play it at all (Evans-Thirlwell, 2016). By comparison, *Spec Ops: The Line* was widely praised for its organic use of moral conflict, with some critics ranking the game among the top video games in the history of the medium precisely due to its morally complex storytelling (Nix, 2020). To this end, Wells (2016) suggests that advances in the narrative design of video games might shift toward experiences in which “violence in games may begin to be recognized as art, rather than considered elements of controversy and concern” (para. 15). Notably, others have demonstrated that heavy engagement with video games featuring gun violence can result in scenario responses (e.g., reactions to images of a gun threat) similar to individuals suffering from post-traumatic stress syndrome (Santos et al., 2019). Thus, it is important to assess ones’ prior exposure to violence broadly, as well as other media violence exposures (see Gerbner, 1980).

### Video Games as Reflective Spaces

In these example games above, game developers are drawing from more established media forms to reconsider the range of reactions they can evoke in players. For example, war films commonly use highly realistic and even graphic violence as part of more serious and somber anti-war messaging (Gates, 2005). Oliver and Raney (2011) explain that films (and entertainment media, broadly) can be understood through two distinctyet-correlated processes: enjoyment, rooted in more hedonic reactions to media content (such as arousal, fun, and pleasure); and appreciation, rooted in more eudaimonic reactions (such as introspection, self-reflection, and poignancy). Oliver et al. (2018) further developed the notion of self-transcendence as a more specific type of media appreciation tied to an emotional and personal growth concerned with contemplation and moral beauty. Broadly speaking, this dual process model of media entertainment has enjoyed a good deal of academic attention in that it helps understand a wider set of audience reactions to media content—including violent media content—that move beyond enjoyment and titillation. Video games in particular are deeply emotional experiences in which players likely experience a circumplex of emotions in direct response to their actions and witnessing the consequences of those actions, as well as pondering those actions in the “real world” (Hemenover and Bowman, 2018).

This expansion of scholarship has included the seemingly paradoxical claim that interactive violence in video games could encourage prosocial reactions in players (Limperos et al., 2013). In an online survey of adults with extensive video gaming experience, Oliver et al. (2015) reported that while nearly all respondents could recall enjoyable responses to gaming content, nearly three in four respondents (72%) were able to discuss appreciation responses; follow-up analysis by Rogers et al. (2016) found that gamers discussing enjoyable or meaningful reactions to video games often mentioned the same video game titles, or games from the same gaming genres—including unexpected sources of appreciation from violent first- and third-person shooters (including *Spec Ops: The Line* and *Call of Duty 2: Modern Warfare* mentioned earlier). Holl et al. (2020) reported similar moral deliberations among a set of experienced gamers who often felt that games can commonly include feelings beyond “just having fun” (p. 3)— unexpected when we consider gaming is often assumed to be more light-hearted and less serious (Bowman et al., 2018). One interpretation of these is that adults who play video games on a regular basis can understand more nuanced portrayals of violence on more contemplative, serious, and humanistic terms. Notably, these studies mostly include convenience samples of adult populations who are experienced gamers and have not yet considered specific personological variables such as emotional intelligence or empathy. Future research into more specific populations—including as populations at high risk of enacting or experiencing interpersonal violence—is warranted.

Experimental data focused on feelings of guilt have shown that when players are forced to commit acts of unjustified violence, post-gameplay guilt reactions are increased (Hartmann et al., 2010; Grizzard et al., 2014). Gollwitzer and Melzer (2012) found that when players performed in-game violence, they engaged in moral cleansing practices such as using hand sanitizer after gameplay. Research into player’s moral decision making generally shows that player’s chronic and established moral sensitivities (e.g., those moral intuitions which guide decision making in everyday life; Haidt and Joseph, 2004) influence player’s in-game choices (Joeckel et al., 2012; Weaver and Lewis, 2012; Boyan et al., 2015). More promising for the study of violence prevention, Tamborini et al. (2018) found decisions to protect in-game others and treat in-game characters fairly were predicted by both chronic and temporary morality—the latter being driven by specific narrative cues. Using modifications to the role-playing video game *Neverwinter Nights 2*, players were asked to run errands for an elderly character and assist villagers with numerous tasks, with each task involving a potential moral violation (such as getting into a physical fight with a tavern owner or stealing money from laborers). After gameplay, players (mostly college-aged students) reported an increased salience toward care and fairness. Recent analyses of players’ unsolicited discussion about video games finds that when acts are explicitly framed as moral dilemmas (such as the *Call of Duty: Modern Warfare 2* scenario discussed earlier), players turned to public spheres such as discussion boards on gaming review pages to debate the morality of their in-game actions (Malazita and Jenkins, 2017). With exception of Malazita and Jenkins (2017), these studies mostly examine college-aged students unlikely to represent a broader spectrum of developmental stages and thus, there is a broad need for replication and extension of this work to consider more diverse populations—even more relevant given claims that gaming experiences are increasingly ubiquitous (Bogost, 2011). To give a rather specific example of emerging research into specific gaming populations, there is a growing body of research on combat veterans using video games as a coping mechanism for post-traumatic stress disorder (PTSD; see Banks and Cole, 2016; Colder Carras et al., 2018), including violent and military-themed first-person shooters (Elliott et al., 2015; Etter et al., 2017). This work is comparatively nascent in the broader literature on violent video games, early results suggest that rather than serving as triggers of PTSD, these games served both short-term (mood management and stress reduction) and long-term (well-being and socialization) psychological outcomes, although veterans also expressed concerns about maladaptive coping (such as playing excessively; Colder Carras et al., 2018).

Finally, players could also reflect on violent acts depending on their relationship to the many characters within a given game, including their own in-game avatar or character. As suggested by Banks (2015), these player-avatar relationships can be understood on a continuum from asocial (in which the player sees the avatar as a mere object for gameplay, void of any emotional attachment) to fully social experiences (in which the player sees the avatar as distinct and authentic social other). Although yet to be tested empirically, these different types of relationships could influence how players respond to interactive media violence, both in terms of how players feel about perpetrating this violence and how they feel about their avatar being the victim of the violence. For example, players with an asocial orientation toward their avatars might not process violent content as anything more than an amoral and distal consequence of gameplay and thus, are unlikely to critically evaluate violent content; at least one study found that players who feel detached from their game characters are more likely to engage in antisocial gameplay patterns (such as challenging or harassing other players; Bowman et al., 2012). By contrast, players adopting a more social orientation are likely to empathize with an avatar that is being victimized by violence (even intervening on the avatar’s behalf), or they might critique an avatar that is perpetrating violence (such as acting to prevent the perpetration; Bowman and Banks, 2021). As a comparatively new area of research, left unresolved are details as to the player-side and game-side variables that encourage these relationships to form. For example, although we might expect different video game genres to encourage some player-avatar relationships over others (e.g., role-playing games to encourage more social relations), Bowman et al. (2016) found no evidence that relationships varied as a function of game type. Related to this, research has yet to understand if and when player-avatar relationships might change over time, or how developmental stages might influence both (a) the types of relationships that people form with their avatars and (b) the impact of those relationships beyond gameplay. Banks (2015) did find that individuals dealing with trauma (such as domestic abuse and issues of gender identity conflict) tended to engage their avatars in a symbiotic capacity—playing themselves in the shoes of the avatar, but crafting an avatar with aspirational or coping elements (such as weapons for strength or banners for identity). Such findings might suggest a capacity for player-avatar relationships to serve both as coping mechanisms for interpersonal violence as well as indicators than an individual is experiencing the same.

To this point, we have intentionally not discussed serious games and simulation gaming, which can be defined by their purpose-driven design (e.g., video games designed with the specific purpose of encouraging prosocial behaviors; see Susi et al., 2007; Ritterfeld et al., 2009)—for example, video games with more prosocial themes can encourage prosocial outcomes (Gentile et al., 2009; Greitemeyer and Mügge, 2014), but those games are usually marked by an absence of violent content (considered anathema to prosocial outcomes). Likewise, we have not discussed the impact of video gaming broadly on cognitive and emotional abilities likely associated with reducing interpersonal violence (for cognitive effects see Bediou et al., 2018; for emotional effects see Hemenover and Bowman, 2018). Both are critical areas of concern that likely help us understand video gaming’s impact on interpersonal violence, deserving of their own discussions. Our claim here is more basic: that we consider more seriously the seemingly paradoxical claim that violent game content might “encourage critical engagement with real world issues and problems, including forms of violence” (Parks, 2009, p. 90).

## VIRTUAL REALITY AND VIOLENCE

Immersive virtual environments, popularly known as virtual reality (VR), are mediated environments created with digital devices that present rich layers of sensory information so that users may see, hear, and feel as if they are in the physical world (Sutherland, 1965). In addition to richer arrays of sensory information, VR extends the user’s ability to interact with the mediated environment through high fidelity, full-body tracking—every movement that the user makes is tracked and rendered rapidly so that the human sensory channels perceive the refreshed and re-rendered virtual worlds as real-time updates. To the end user, virtual experiences in VR feel as authentic as experiences in the physical world.

This experience of users feeling as if they have visited the mediated world—the illusion of the experience feeling so authentic that the user perceives it to be a non-mediated event—is referred to as *presence* (Slater and Usoh, 1993; Biocca, 1997; Lombard and Ditton, 1997). Presence is perceived when stimuli from the virtual world progressively occupy users’ sensory channels to a level sufficient to evoke the perception that the mediated stimuli are genuine (Biocca, 1997). VR experiences tend to elicit a higher level of presence perception than media experiences through more traditional platforms (Sallnäs, 2005; Persky and Blascovich, 2007; Ahn et al., 2016; Cummings and Bailenson, 2016). These findings suggest that experiences in VR better mimic firsthand experiences in the physical world than any other existing platform.

To date, very little work in VR has looked directly at violence or violence prevention, and some of this work is conflated with a focus on VR video games (Calvert and Tan, 1994; Persky and Blascovich, 2007). This is likely due to a broad inaccessibility of VR systems and a general lack of violent content relative to widely available video games. However, this is changing rapidly with the introduction and adoption of accessible and affordable consumer grade VR devices and an accompanied growth of content, some of which can depict violence with rich layers of sensory information and contextual details (Gonzalez-Liencres et al., 2020). For instance, newer virtual experiences allow users to experience firsthand the gruesome reality of surviving in a warzone (e.g., *The Fight for Falluja*) or living life as a refugee (e.g., *Clouds Over Sidra*). However, these VR experiences differ from video games (including games of similar content, such as the aforementioned *Spec Ops: The Line*) in that they often lack a specific goal as well as common video game mechanisms, such as points, badges, or leaderboards. Unlike video games, VR presents experiences that are meant to be lived rather than played.

Therefore, extending the limited early work on violence in VR and how it relates to user experiences both in and outside of the virtual world is a critical and timely question to address. The growing body of relevant research in VR, albeit not directly investigating violence, may also provide insights for inferences to be made. These insights may not offer immediate and definitive answers to how VR should be used to prevent violence in the physical world but may motivate future research by highlighting the connections between extant scholarship in VR and violence prevention research. The non-violent VR experiences may also be applied to primary prevention efforts to improve general user skills that could help prevent violence in the future.

### Virtual Experiences Impact Physical Behaviors

People learn from both direct and indirect experiences. Bandura (1986) details how humans generally rely on their cognitive abilities to symbolize external environments and the events that take place within. Bandura argues that this ability to create cognitive models of the world based on symbolization and abstraction allows people to understand and process indirect, vicarious experiences. Accordingly, decades of mass media research have demonstrated that the impact of mass media message consumption leads to real world outcomes, ranging from health behavior changes (Wakefield et al., 2010), shifts in attitudes toward social issues (McLeod and Detenber, 1999), and learning (Papa et al., 2000). VR contributes another layer of complexity in the user-media relationship by providing users with a highly interactive environment in which users become the agent of their own media experiences. Users have high agency in VR, controlling the field of view, manipulating objects, and locomoting through the mediated space at will, blurring the boundaries between content producer and consumer. Thus, virtual experiences are better able to mimic direct, firsthand experiences than traditional media (Blascovich and Bailenson, 2011). Individuals place greater weight on direct, rather than indirect, experiences when making decisions, and consequently, direct experiences tend to have stronger and longer lasting impact on attitude changes than indirect experiences (Fazio and Zanna, 1981).

Perhaps one of the most critical opportunities that VR provides for the primary prevention of violence is the fact that the impact of experiences in VR does not end when the user “unplugs” and leaves the virtual world; rather, the effects transfer into the physical world to shift the user’s attitudes and behaviors, such as adopting recommended health attitudes and behaviors in the domains of eating (Ahn, 2015), vaccination (Nowak et al., 2020), exercising (Fox and Bailenson, 2009), adopting pro-environmental behaviors (Ahn et al., 2014), and helping others (Ahn et al., 2013; Rosenberg et al., 2013) in the physical world. A growing number of studies demonstrate that users temporarily shift the attitudes and behaviors of their physical selves to match those of their virtual selves (Yee and Bailenson, 2007; Ratan et al., 2018). Compared to traditional platforms, the magnitude of these changes is stronger and lasts longer over time (Ahn, 2015; Ahn et al., 2015; Herrera et al., 2018). Counter to what intuition might suggest, virtual experiences are not transient and virtual interactions are not intangible.

VR systems have become dramatically more affordable and user-friendly. For example, Facebook’s Oculus Quest system has eliminated the need for separate tracking cameras, wires, controllers, or even computers to immerse users in virtual worlds. These self-contained or “stand-alone” systems are usually less expensive (the Quest 2 will retail at $299). These advancements have brought forth a renewed interest in social VR, where large numbers of users can simultaneously meet and interact in VR (Schroeder, 2010). Although formal scientific studies have yet to rigorously test the impact of social VR on interactions in the physical world, anecdotal stories abound of people attempting to resolve problems within virtual relationships in the physical world (e.g., adultery online leading to confrontations and even divorces offline; Craft, 2012)—stories not so unique from the earliest history of social networking technologies such as bulletin board systems and text-based chat rooms (see Malloy, 2016).

The impact of virtual experiences on physical world attitudes and behaviors pose an interesting complexity to using VR as a tool for violence prevention. Based on the aforementioned findings that effects of virtual experiences transfer into the physical world to impact attitudes and behaviors, one aspect to consider is that violence experienced in the virtual world is likely to affect ensuing experiences in the physical world. Therefore, when integrating elements of violence exposure as a part of the intervention, individuals should also be trained to be cognizant that the impact of being exposed to virtual violence (both as a perpetrator and a victim) may not dissipate immediately upon leaving the virtual world. Consideration should also be put forth regarding potential psychophysiological duress that individuals may experience when being exposed to virtual violence. Earlier research has demonstrated that when asked to apply electric shocks to an avatar, participants displayed psychophysiological responses as if they were applying shocks to a real person, even when they were well aware that neither the avatar nor the electric shocks were real (Gonzalez-Franco et al., 2018). These earlier findings are notable particularly when considering that these virtual experiences are not designed to entertain but rather are crafted as simulations, and unlike many video games, may be perceived as an authentic firsthand experience rather than entertainment. The experience of violence either as entertainment or non-entertainment may be an important point of distinction, particularly in terms of their impact on physical attitudes and behaviors. A growing body of literature notes that audience views on violence in entertainment media are complicated, involving entertainment elements such as the likeability of the villain, feelings of justice restored, and elation at watching survivors, which has been shown to increase audiences’ enjoyment of violent content (Oliver and Sanders, 2004). When stripped of these entertaining elements, scholars have posited that the seriousness and gravity of violent content may be highlighted (Rovira et al., 2009). Violent experiences perceived as valid and plausible events are likely to have stronger and more persistent impacts that transfer into the physical world than violence experienced as mere entertainment.

### Reducing Emotional Trauma Through Virtual Experiences

Because events in virtual worlds are perceived as authentic, firsthand experiences that continue to affect users after they have left the virtual world, violence prevention research in the context of VR must consider prevention efforts for both within and outside of the virtual world. The rich layers of sensory information may render violence in VR to feel comparable to violence experienced in the physical world in terms of its emotional intensity, and its negative consequences may possibly transfer over into the physical world to interfere with the victim’s life after the virtual experience has ended. This is a critical takeaway because it underscores the importance of coupling knowledge from traditional violence prevention interventions with knowledge about these emerging technologies. Because the virtual and physical worlds are closely intertwined—experiences in one world impacting experiences in the other—leveraging core strategies to prevent violence in the physical world and augmenting them through novel features of VR would serve as comprehensive and creative solutions for violence prevention.

However, when designed carefully by experts and integrated with existing treatment protocols, there is strong evidence that controlled exposure to negative or traumatic events can help address psychosocial disorders, such as post-traumatic stress disorders (PTSD; Foa et al., 2007), phobias (Craske et al., 2008), and body image related disorders (Rosen et al., 1995). For example, in exposure therapy for PTSD, desensitization to traumatic memories by reliving parts of the experience is critical to facilitate emotional process. These findings have yet to be tested with diverse populations so generalizations should be made tentatively, but earlier results demonstrate the promise that VR platforms hold in allowing therapists to recreate experiences that may be difficult, impossible, or fatal in the physical world. With full control over all aspects of the virtual experience, therapists can work with patients to tolerate the exposure to the feared or traumatic element and gradually habituate and desensitize emotional responses toward the stimuli (Ready et al., 2006). Furthermore, using VR in therapy is anticipated to increase face validity of the treatment, and when combined with traditional treatment (such as cognitive behavioral therapy or medication), patients’ overall treatment time is reduced, which is anticipated to help increase compliance with treatment protocols (Difede et al., 2019).

The success in incorporating VR for exposure therapy might have meaningful implications for violence prevention efforts. Given that perpetrators of violence are sometimes themselves victims of violence, particularly in early childhood (Hughes et al., 2017), safe, stable, nurturing relationships, and environments are critically important for violence prevention (Merrick et al., 2013). Because researchers and clinicians have full control over the virtual experience and the content that users are exposed to, VR, with the supervision of a trained clinician, provides a relatively safe and controlled environment for individuals who have been exposed to violence, and therefore have an increased likelihood to become violent to others, to confront their trauma at their own pace. The effect of the training that takes place in the virtual world is then anticipated to carry over into the physical world to assist individuals in diffusing situations that they may have reacted violently to without the intervention.

### Embodying Experiences of Victims, Perpetrators, and Bystanders

Taking the perspective of others has been proven to facilitate social interaction and communication by establishing common grounds between interactants so that they may infer shared knowledge and beliefs (Krauss and Fussell, 1991). Sharing the same basis of feelings and thoughts with another person encourages mutual understanding and helping behaviors (Batson, 1991; de Waal, 2008). For this reason, perspective-taking and role playing have played central roles in violence prevention efforts—including programs such as the “Green Dot” (Coker et al., 2017) and “Bringing in the Bystander” (Banyard et al., 2007; Edwards et al., 2019). However, perspective-taking is a controlled, effortful process that requires substantial cognitive resources and can be challenging for individuals who may be mentally fatigued or lack the motivation to invest the effort (Klein and Hodges, 2001; Gehlbach et al., 2012). Furthermore, engaging in role-playing without contextual details of the violent event (e.g., where the event took place, the ambient sounds, who was there) may be insufficient in delivering the urgency or gravity of the situation and individuals are likely to perceive the role-playing exercise as a mere formality (Jouriles et al., 2009, 2011).

In response, VR systems provide rich, multilayer perceptual information and create embodied experiences so that users are able to see, hear, and feel as if they have become another person. Individuals can be placed in the heat of the moment of the violent event as if it were happening to them and experience the same event as perpetrators, victims, or bystanders at the click of a button. For example, albeit in the context of a nonviolence experience, using VR to take the perspective of another person with a disability in the virtual world increased feelings of psychological merging between interactants to reduce negative attitudes and biases against persons with disabilities (Ahn et al., 2013). More importantly, the effects of sharing the “lived” experiences of persons in need, who are struggling with physical disabilities or circumstances such as social inequality, transferred into the physical world to increase helping behavior over time (Ahn et al., 2013; Herrera et al., 2018).

More relevant to the context of violence prevention, Seinfeld et al. (2018) found preliminary evidence that males with a history of domestic violence had more trouble recognizing fear in female faces than males without a history of violence. When the males with a history of domestic violence embodied the experience of a female victim in VR, their ability to recognize fear in female faces improved and their tendency toward associating fearful female faces as happy was reduced. Similarly, de Borst et al. (2020) reported that men who experienced a domestic violence incident in VR in the first-person perspective activated neural networks associated with feelings of identification for a virtual victim, even when they had never experienced domestic violence in the physical world. VR’s ability to construct the common ground of understanding victims’ experiences from their perspective may assist efforts for violence prevention among a broader audience, including those who may not have prior exposure to violence and, as a result, fail to understand the critical elements of preventing and dealing with violence. Likewise, Jouriles et al. (2009, 2011) validated and demonstrated that role-playing in VR is more effective than traditional role-playing methods often adopted in counseling and training for violent events, because of the realism of contextual details and the sense of presence that the virtual experience offers.

Earlier research in bystander interventions notes that bystanders often fail to recognize a bullying situation taking place in front of them, particularly when victims are being exposed to covert and tacit violence (Killer et al., 2019). Moreover, cyberbullying perpetrators may not recognize the added pressure on the victims who are unable to get a reprieve from the bullying in a constantly connected world, which could hinder their ability to empathize with victims. This ability to view and live through the same experience from different perspectives is likely to allow all parties involved to understand the complexities involved in a violent event—violence may be perceived very differently when it is experienced as a victim, perpetrator, or bystander. VR ability to demonstrate this difference using the same violence event may facilitate conversations between patients and clinicians, and further research is necessary to provide empirical support. Finally, Sargent et al. (2020) also demonstrated that VR may be used to objectively and unobtrusively assess bystander behaviors by logging user behaviors during their engagement with the VR experience that simulated violent events such as physical dating violence, stalking, or coercive relationships. User responses in VR involving peer pressure resistance (ability to resist pressure from avatars controlled by actors) and bystander responses (effectiveness of user intervention in risky situations) were coded and validated to demonstrate that VR may also serve as a psychometrically sound addition to self-reports to assess responses to violent events.

### Experiencing Future Benefits of Violence Prevention

Future orientation is an individual’s tendency to think about the future and plan ahead before acting by anticipating future consequences (Trommsdorff, 1983). Having a positive future orientation toward life motivates individuals to engage in less compromising behaviors and promotes behaviors that would help them move toward their vision of the future (Arnett, 2000). However, maintaining an orientation toward the future is not always easy, particularly when presented with attractive options in the present. The temporal delay between present day choices and future consequences can render the causal relationship abstract and selecting present day behaviors for delayed gratifications in the future can be challenging for many.

Some research has demonstrated that encouraging future orientation in adolescents so that they can consider negative consequences of engaging in violent behavior and envision a future where they have successfully met their life goals is effective in reducing violence (Stoddard et al., 2011). A growing collection of research demonstrates that VR can effectively demonstrate future negative consequences of present behaviors, thereby promoting favorable health behaviors (Fox and Bailenson, 2009; Persky and McBride, 2009; Ahn, 2015), and similar approaches have been successful in promoting pro-environmental behaviors (Zaalberg and Midden, 2013; Ahn et al., 2014, 2016), where future oriented thinking has been shown to demonstrably increase risk perceptions (Lee et al., 2020). These earlier findings suggest promising potentials for using VR to prevent violence by having individuals live through future negative consequences as if they were happening at the moment.

The idea of using future orientation to modify present behaviors is not new. Literature from multiple disciplines have documented how individuals struggle to make intertemporal choices, in which decisions must be made for benefits that occur now vs. benefits for the future (Schelling, 1982; Laibson, 1997). Because people generally place greater priority to benefits in the present while caring less about benefits in the future (temporal discounting, see Chapman, 1996), scholars have had difficulty persuading them to change their present day behaviors for future benefits. The fundamental reason behind this struggle seems to be because people consider the future self as someone disconnected to the present self (seeing the future self as a stranger; Pronin and Ross, 2006), when the events that take place in the extremely distant future seem abstract and irrelevant to present events. In VR, users embody events set in the future so that the events feel as if they are happening at the moment. Studies have demonstrated that these embodied experiences lead to feelings of urgency and immediacy among users (Ahn, 2015; Ahn et al., 2016) and an increased sense of connection between the present and future selves (Hershfield et al., 2011).

## DISCUSSION AND FUTURE RESEARCH DIRECTIONS

Given the ubiquity of video gaming, increased access to VR, and myriad forms of content in both technologies, interactive media violence likely exist at and operate in all levels of the social ecological model. At the individual level, experiences with video games and VR can foster both cognitive skills (such as information processing, Green, 2018) and emotional skills (such as emotional regulation, Hemenover and Bowman, 2018) that might serve as protective factors for violence prevention. One area of future research may consider the influence of adverse childhood experiences on how one processes and responds to interactive media violence. At the relational level, a core feature of video games and VR is their sociality (especially online games, see Steinkuehler and Williams, 2006) and potential for reciprocity among players (Velez et al., 2016). Notably, social connectedness (e.g., peer relationships) is a violence protective factor, and future research may examine the role that interactive media violence plays within and between various peer groups, both online and in person. For example, relational-level efforts could use interactive media violence to organize and facilitate interactions and conversations among the many shareholders affected by interpersonal violence—for example, serving as robust and powerful experiences to communicate risks to potential perpetrators and share victims stories in authentic and meaningful ways. Video game technologies might provide comparatively safe fantasy spaces to better understand the dynamics of interpersonal violence, while VR technologies can quite literally allow for shared experiences of the violent event that can be seen, heard, and felt from both the perspective of the victim or the perpetrator. At the community and societal levels, future research exploring where and how interactive media violence is engaged with and discussed by institutions—from schools and community centers to larger media systems—could be critical in understanding whether or not such content is accepted as an alternative means for violence prevention (similar to how films such as “Schindler’s List” or “Hotel Rwanda” might be screened), or relegated to moral panic status (Bowman, 2016). At a macro level, violence in myriad forms (including interpersonal violence) is already part of video games and VR and as such, it is unrealistic to presume that all such content can be avoided or eliminated from these spaces. Discussions of and exposure to such content may be fostered under the guidance of peers, parents, teachers, trained clinicians, and others allowing for a more proactive and upstream (re: primary prevention) approach to preventing deleterious effects such as interpersonal violence (Centers for Disease Control and Prevention, 2020b). Participatory design principles could also be considered as way mitigate harmful content, and likewise content in both spaces can be and is monitored through shared use and behavior policies to prevent interpersonal violence online (such as cyberbullying). Broadly, future work is needed to better understand the impacts of interactive media violence at all levels of the social ecology, as they can be informative to violence prevention public health approaches.

Understanding the influence of interactive media violence on aggressive and violent outcomes has been a critical concern of recent media effects research. Extant literature has consistently shown a small-yet-statistically significant association between violent media and aggressive outcomes, and these effects also include interactive media violence. Yet as video games and VR technologies become increasingly more complex and diverse—both in terms of technical proficiency and narrative complexity—there are numerous opportunities to examine the potential for these digital and interactive experiences to support violence prevention. Emerging evidence from media psychology and related fields suggests the possibility that for some users, experiences with on-screen (or in-headset) violence can influence how we think about, feel toward, and react to interpersonal violence. Given the limited scope of this research, the dynamic nature of interactive media development, and the known risks associated with violent content on aggression, research is needed to understand how interactive media may foster and influence protective factors or possibly interventions for preventing real-world violence.
